# Kinetic Spectrofluorometric Determination of Certain Calcium Channel Blockers via Oxidation with Cerium (IV) in Pharmaceutical Preparations

**Published:** 2009-06

**Authors:** M. I. Walash, F. Belal, N. El-Enany, A. A. Abdelal

**Affiliations:** *Department of Analytical Chemistry, Faculty of Pharmacy, University of Mansoura, Mansoura, Egypt*

**Keywords:** verapamil hydrochloride, diltiazem hydrochloride, nicardipine hydrochloride, Cerium (IV)

## Abstract

A simple and sensitive kinetic spectrofluorometric method was developed for the determination of some calcium channel blockers namely, verapamil hydrochloride, diltiazem hydrochloride, nicardipine hydrochloride and flunarizine. The method is based upon oxidation of the studied drugs with cerium (IV) ammonium sulphate in acidic medium. The fluorescence of the produced Ce (III) was measured at 365 nm after excitation at 255 nm. The different experimental parameters affecting the development and stability of the reaction product were carefully studied and optimized. The fluorescence-concentration plots were rectilinear for all the studied compounds over the concentration range of 0.01 to 0.12 μg mL^-1^. The limits of detections for the studied compounds ranged from 2.93 × 10^-3^ to 0.012 μg mL^-1^ and limits of quantification from 9.76 × 10^-3^ to 0.04 μg mL^-1^ were obtained. The method was successfully applied to the analysis of commercial tablets. The results obtained were in good agreement with those obtained with reference methods.

## INTRODUCTION

**Figure 1:**
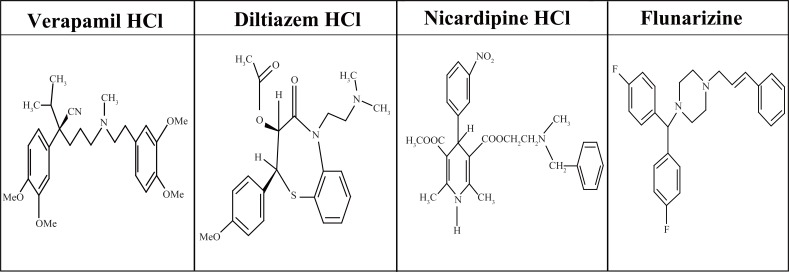
Structural formulae of the studied drugs.

Verapamil hydrochloride (VP), diltiazem hydrochloride (DLT), nicardipine hydrochloride (NC) and flunarizine (FZ) (Fig. 1) are widely used calcium channel blockers. VP, DLT and NC are currently used for the management of angina pectoris, and also used in the treatment of hypertension. As for FZ, it is used for migraine prophylaxis, for vertigo and vestibular disorders and for peripheral and cerebral vascular disorders (1).

Several analytical methods have been reported for the determination of VP either per se or in pharmaceutical preparations including: spectrophotometry (2–4), fluorometry (5, 6), voltammetry (7), HPLC (8-13) and capillary electrophoresis (14). VP is the subject of a monograph both of the British Pharmacopoeia, BP (15); and the United State Pharmacopoeia, USP (16). Both the BP and USP recommend non-aqueous titration for the raw material and spectrophotometric measurement at 278 nm for the tablets. The USP (16), on the other hand, recommend HPLC method for its formulations.

Regarding DLT, various methods have been applied for its determination in its formulations such as spectrophotometry (17, 18), voltammetry (19), HPLC (20-23). The BP (15) recommends non-aqueous titration for the raw material, The USP (16), on the other hand, recommend HPLC method for its formulations.

Several methods have been utilized for the quantitative estimation of NC in its pharmaceutical preparations including: spectrophotometry (24, 25), voltammetry (26, 27), HPLC (28-31) and capillary electrophoresis (32).

Relatively few methods have been described for the determination of FZ in its formulations viz spectrophotometry (33, 34), fluorometry (35), voltammetry (36) and HPLC (37-39).

The proposed method depends simply on oxidation of all the studied drugs with Ce (IV) in acidic medium and measuring the intensity of the formed Ce (III) at 365 nm after excitation at 255 nm. Compared with the reported spectrofluorimetric methods (5, 6) for VP, the proposed method is more sensitive, since the working concentration range of the reported methods ranged from 1-10 μg mL^-1^ (5). In the other method, the determination was conducted on 1 μg mL^-1^ with detection limit in the range of 0.04-0.1 μg mL^-1^ (6). However, the proposed method is highly sensitive, it could measure as low as 0.01-0.12 μg mL^-1^. Similarly, for FZ, the working concentration range of the reported methods ranged from 0.94-7.1 μg mL^-1^ (35), and poor sensitivity compared with the proposed method.

To the best of our knowledge, no spectrofluorometric method has been reported for the analysis of DLT and NC up till now. This initiated the present study.

## EXPERIMENTAL

### Apparatus

The fluorescence spectra and measurements were recorded using a Perkin-Elmer UK model LS 45 luminescence Spectrometer, equipped with a 150 Watt Xenon arc lamp, gratting excitation and emission monochromators for all measurements and a Perkin-Elmer recorder. Slit widths for both monochromators were set at 10 nm. A 1 cm quartz cell was used.

### Materials and Reagents

All reagents and solvents were of Analytical Reagent Grade.

- Verapamil hydrochloride, diltiazem hydrochloride, nicardipine hydrochloride and flunarizine pure samples were purchased from Sigma (St. Louis, Mo, USA) and used as received.
- Cerium (IV) ammonium sulphate, (BDH, Pool, UK), 5 × 10^-4^ M aqueous solution was freshly prepared in 1.0, 1.25 and 1.5 M sulphuric acid.
- Sulphuric acid, (Prolabo, France), 1.0, 1.25 and 1.5 M aqueous solutions.

### Standard Solutions

Stock solutions of VP, DLT and NC were prepared by dissolving 10.0 mg of each of the studied compounds in 100.0 mL of distilled water, while stock solution of FZ was prepared by dissolving 10.0 mg of FZ in 100.0 mL of 2 M H_2_SO_4_ solution, and was further diluted with the same solvent as appropriate. The standard solutions were stable for 10 days when kept in the refrigerator.

### General Procedures

Aliquot volumes of VP, DLT, NC and FZ standard solutions covering the working concentration range cited in Table 1 were transferred into a series of 10 mL volumetric flasks; followed by specified volumes of 5 × 10^-4^ M Ce (IV) solution as shown in Table 1. The flasks were heated in a thermostatically controlled water-bath at 100 °C for specified time (Table 1). The solutions were cooled and diluted to the mark with distilled water. A blank experiment was performed simultaneously. The relative fluorescence intensity (FI) of the solutions was measured at 365 nm after excitation at 255 nm. The corrected FI was plotted vs final concentration of the drug (μg mL^-1^) to get the calibration graphs. Alternatively, the corresponding regression equations were derived.

**Table 1:** Performance data of the proposed method

Parameter	Verapamil HCl	Diltiazem HCl	Nicardipine HCl	Flunarizine

Concentration range (μg mL^-1^)	0.02-0.12	0.01-0.06	0.02-0.12	0.04-0.12
Volume of 5 × 10^-4^ M Ce (IV), mL	0.5	1	1.5	0.8
Concentration of H_2_SO_4_ (M)	1.25	1	1.5	1.5
Time of heating (min)	25	20	15	25
Limit of detection (LOD) (μg mL^-1^)	6.33 × 10^-3^	3.1 × 10^-3^	2.93 × 10^-3^	0.012
Limit of quantification (LOQ) (μg mL^-1^)	0.02	0.01	9.76 × 10^-3^	0.04
Correlation coefficient (r)	0.9998	0.9998	0.9999	0.9998
Slope	4260.00	7380.233	2992.857	3340.00
Intercept	7.133	9.512	3.670	4.60
S_y/x_	3.110	3.210	0.344	2.530
S_a_	26.750	32.100	2.924	26.820
S_b_	37.020	32.380	4.095	40.000
RSD (%)	1.252	1.220	0.260	1.020
% Error	0.511	0.550	0.106	0.460
Student’s *t* test	1.19	0.28	1.47	0.11
F test	3.24	1.14	2.96	1.48

S_y/x_, Standard deviation of the residuals; S_a_, Standard deviation of the intercept; S_b_, Standard deviation of the slope; % Error, %RSD/√n; RSD% = Relative standard deviation.

## APPLICATIONS

### Procedure for dosage forms

An accurately weighed quantity of the mixed contents of 10 capsules or powdered tablets equivalent to 10.0 mg of VP, DLT and NC was transferred into small conical flask and extracted with 3 × 30 mL of distilled water while FZ capsules were extracted with 3 × 30 mL of 2 M H_2_SO_4_ solution. The extract was filtered into 100 mL volumetric flask. The conical flask was washed with few mLs of distilled water. The washings were passed into the same volumetric flask and completed to the mark with the same solvent. Aliquots covering the working concentration range cited in Table 1 were transferred into 10 mL volumetric flasks. The “General Procedures” were then applied and the nominal content of capsules or tablets was determined either from a previously plotted calibration graph or from the corresponding regression equation.

## RESULTS AND DISCUSSION

**Figure 2:**
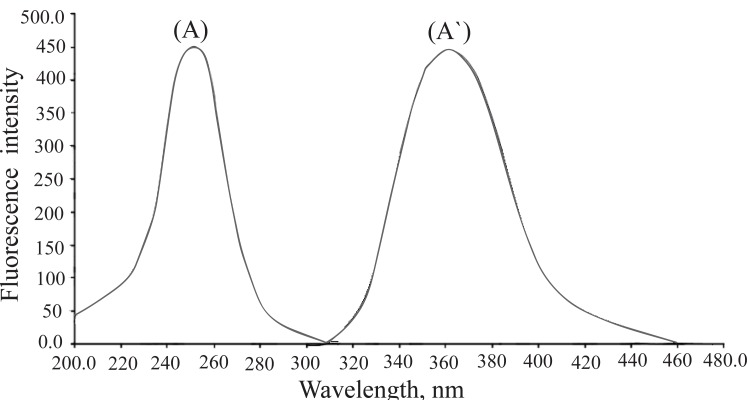
Excitation and emission spectra of induced Ce(III) by oxidation of 0.06 μg mL^-1^ DLT with Ce (IV) where: (A), Excitation spectrum; (A`), Emission spectrum.

Recently, ceric (IV) has been frequently utilized as a useful reagent for the determination of pharmaceutical compounds, such as antivirals (41), some psychoactive drugs (42), aztreonam (43) and isoxsuprine hydrochloride (44). As the fluorescence intensity of the formed Ce (III) increases with the time, this fact was used as a basis for a useful kinetic method for the quantitative determination of certain calcium channel blockers in pharmaceuticals. In the present study, oxidation of the studied drugs with Ce (IV) in an acid medium yields an equivalent amount of fluorescent Ce (III) which exhibits maximum fluorescence at 365 nm after excitation at 255 nm. Figure 2 illustrates the resulting fluorescence spectra of the produced Ce (III) in an acidic medium. The oxidation products were found to be non fluorescent product. This confirmed the fluorescence induced in the oxidation of the investigated drugs with Ce (IV) was not attributed to their oxidation products; however, it was mainly due to the formation of Ce (III).

## OPTIMIZATION OF EXPERIMENTAL CONDITIONS

The spectrofluorometric properties of Ce (III) as well as the different experimental parameters affecting its formation and its stability were carefully studied and optimized. Such factors were changed individually while the others were kept constant. These factors included the Ce (IV) concentration, type of acid and its concentration, heating time, temperature and diluting solvents.

### Effect of Ce (IV) concentration

**Figure 3:**
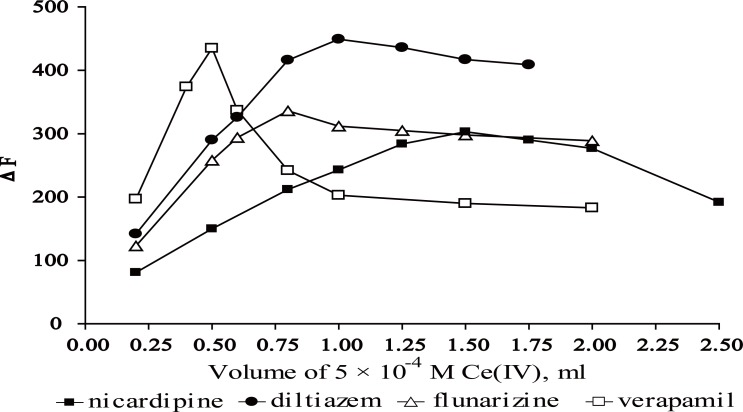
Effect of volume of 5 × 10^-4^ M Ce (IV) on the relative fluorescence intensity. ●, 0.06 μg mL^-1^ DLT; □, 0.1 μg mL^-1^ VP; Δ, 0.1 μg mL^-1^ FZ; ■, 0.1 μg mL^-1^ NC.

The influence of Ce (IV) volume on the fluorescence intensity of the formed Ce (III) was studied using increasing volumes of 5 × 10^-4^ M Ce (IV) solution. It was found that maximum and constant fluorescence intensity was attained upon using 0.5, 1, 1.5 and 0.8 mL for VP, DLT, NC and FZ, respectively as shown in (Fig. 3) and Table 1.

### Effect of acid type and its concentration

**Figure 4:**
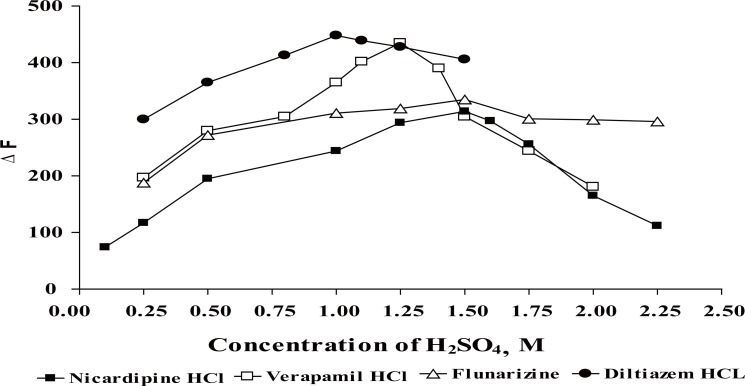
Effect of molar concentration of H_2_SO_4_ on the relative fluorescence intensity. ●, 0.06 μg mL^-1^ DLT; □, 0.1 μg mL^-1^ VP; Δ, 0.1 μg mL^-1^ FZ; ■, 0.1 μg mL^-1^ NC.

The oxidation reaction of Ce (IV) have to be performed in acid medium to prevent precipitation of Ce (OH)_3_. Different acids such as, sulphuric acid, hydrochloric acid, nitric acid and perchloric acid were tested to determine the most suitable one for the reaction. Nitric acid is not preferred to be used owing to the inhibitory effect of nitrate ions on the fluorescence of Ce (III) (44). In the presence of hydrochloric acid, perchloric acid and sulphuric acid, the reaction rate and the fluorescence of Ce (III) were found to be high. However, hydrochloric acid and perchloric acid gave high blank readings, so sulphuric acid was selected for this study. The effect of sulphuric acid concentration on the fluorescence intensity was studied using concentrations ranging from 0.25 to 3 M (Fig. 4). The results are abridged in Table 1.

### Effect of temperature and heating time

**Figure 5:**
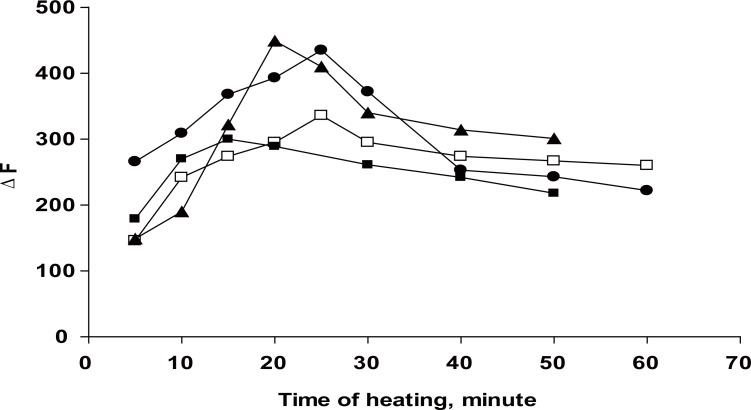
Effect of the heating time on the relative fluorescence intensity. Δ, 0.06 μg mL^-1^ DLT; ●, 0.1 μg mL^-1^ VP; □, 0.1 μg mL^-1^ FZ; ■, 0.1 μg mL^-1^ NC.

Oxidation of the studied drugs with Ce (IV) was carried out at different temperature setting, using a thermostatically controlled water bath, ranged from ambient temperature, 40, 60, 80°C and boiling water bath for period of times ranging from 5 to 60 min. the results are shown in (Fig. 5) and Table 1.

### Effect of diluting solvents

Dilution with different solvents such as water, methanol, acetonitrile, dimethyl sulfoxide and dimethyl formamide was attempted. It was found that, water was the best solvent for dilution as it gave the highest fluorescence intensities and the lowest blank reading, moreover its choice adds to the advantages of the proposed method. Distinct and sharp decrease in the fluorescence intensities was attained upon using acetonitrile and methanol, while dimethyl sulfoxide and dimethyl formamide quenched the fluorescence completely.

## STUDY OF THE KINETIC PARAMETERS

The rate of the reaction was found to be dependent on the concentration of the studied drugs. The rates were followed with various concentrations in the range of 0.02-0.12 μg mL^-1^ for VP and NC, 0.01-0.06 μg mL^-1^ for DLT and 0.04-0.12 μg mL^-1^ for FZ keeping Cerium (IV) and H_2_SO_4_ acid concentrations constant at the recommended levels mentioned before. The rate of the reaction was found to obey the following equation:

(a) $$\text{Rate of the reaction}=\Delta \text{F}/\Delta \text{t}=\text{k}`{\left[\text{drug}\right]}^{\text{n}}$$

where K` is the pseudo-order rate constant and n is the order of the reaction.

The rate of the reaction may be estimated by the variable time method measurement (45), where F is the fluorescence intensity and t is the time in seconds. Taking logarithms of rates and drug concentrations (Table 2), the previous equation is transformed into:

(b) Log (rate)=LogΔF/Δt=Log k`+n Log [drug]

**Figure 6:**
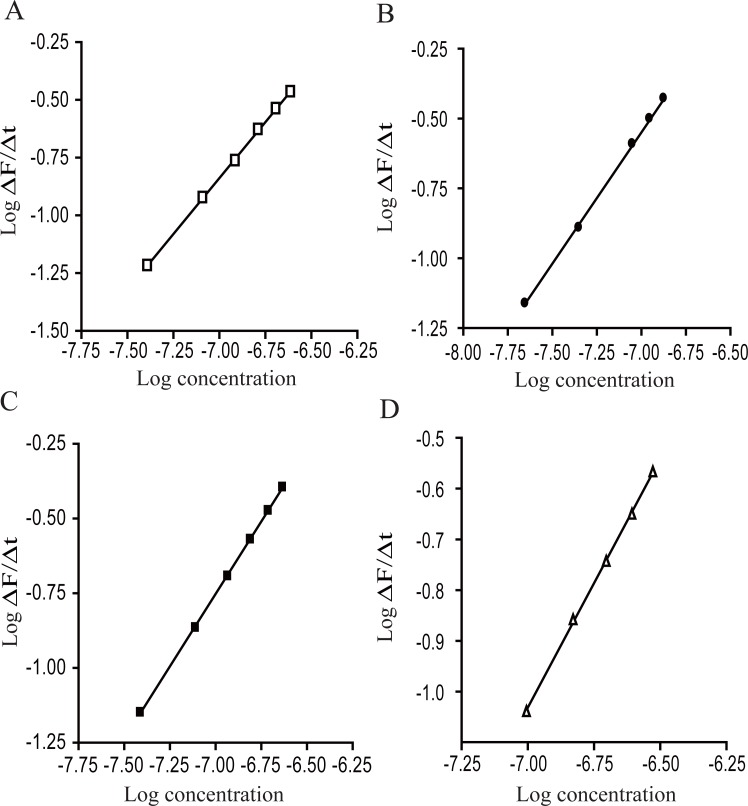
Plot of Log reaction rate (Log ΔF/Δt) versus Log concentration of: A, VP; B, DLT; C, NC; D, FZ.

A plot of log reaction rate versus log concentration of the drug is shown in Figure 6 at 365 nm after excitation at 255 nm, where the slope (n) is the order of the reaction and the intercept (Log K`) is the logarithm of the rate constant (Fig. 6).

A Plot of log reaction rate versus log drug concentration resulted in a pseudo-order rate constant and first order of the reaction which are abridged in Table 2. These results indicated that the reaction is pseudo first order reaction, depending on the drugs concentration.

**Table 2:** Logarithm of the rate for different concentrations of the studied drugs by the proposed method

Compound	Log ΔF/Δt	Log [drug]	Regression equation	Correlation coefficient	Rate constant (S^-1^)	Order of reaction (n)

Verapamil HCl	-1.22	-7.39	Log rate=5.950+0.970 log C	0.9999	891251	0.970
	-0.92	-7.09				
	-0.76	-6.91				
	-0.63	-6.79				
	-0.54	-6.69				
	-0.46	-6.61				
Diltiazem HCl	-1.16	-7.65	Log rate=6.104+0.950 log C	0.9998	1270574	0.950
	-0.89	-7.35				
	-0.59	-7.05				
	-0.50	-6.96				
	-0.43	-6.88				
Nicardipine HCl	-1.15	-7.42	Log rate= 6.037+0.970 log C	0.9999	1088930	0.970
	-0.86	-7.11				
	-0.69	-6.93				
	-0.57	-6.81				
	-0.47	-6.71				
	-0.39	-6.63				
Flunarizine	-1.04	-7.00	Log rate=5.834+0.981 log C	0.9998	682339	0.981
	-0.86	-6.83				
	-0.74	-6.70				
	-0.65	-6.61				
	-0.57	-6.53				

## SELECTION OF THE BEST KINETIC METHOD

Several kinetic techniques were adopted for the selection of the best method. Rate constant, fixed fluorescence and fixed time methods (46, 47) were tried and the most suitable analytical method was selected taking into account the applicability, the sensitivity, i.e. the slope of the calibration graph and the correlation coefficient (r).

### Rate constant method

Graphs of log absorbance versus time for VP, DLT, NC and FZ concentration in the range of 4.07 × 10^-8^ – 2.44 × 10^-7^ M, 2.22 × 10^-8^ – 1.33 × 10^-7^ M, 3.88 × 10^-8^ – 2.33 × 10^-7^ M and 9.89 × 10^-8^ – 2.97 × 10^-7^ M, respectively were plotted and all appeared to be rectilinear. Pseudo-first order rate constants (k`) corresponding to different drug concentrations (C) were calculated from the slopes multiplied by – 2.303 and are presented in Table 3.

**Table 3:** Application of the rate constant method in the quantification of the studied drugs by the proposed method

Compound	k`(S^-1^)	[drug]

Verapamil HCl	-4.190 × 10^-4^	0.41 × 10^-8^
	-4.100 × 10^-4^	0.81 × 10^-8^
	-4.080 × 10^-4^	1.22 × 10^-7^
	-4.053 × 10^-4^	1.63 × 10^-7^
	-4.023 × 10^-4^	2.04 × 10^-7^
	-4.000 × 10^-4^	2.44 × 10^-7^
Diltiazem HCl	-1.283 × 10^-3^	0.22 × 10^-8^
	-1.262 × 10^-3^	0.44 × 10^-8^
	-1.235 × 10^-3^	0.89 × 10^-8^
	-1.215 × 10^-3^	1.11× 10^-7^
	-1.184 × 10^-3^	1.33 × 10^-7^
Nicardipine HCl	-1.263 × 10^-3^	0.39 × 10^-8^
	-1.225 × 10^-3^	0.78 × 10^-8^
	-1.200 × 10^-3^	1.16 × 10^-7^
	-1.176 × 10^-3^	1.55 × 10^-7^
	-1.139× 10^-3^	1.94 × 10^-7^
	-1.124 × 10^-3^	2.33 × 10^-7^
Flunarizine	-6.351 × 10^-4^	0.99× 10^-8^
	-6.296 × 10^-4^	1.48 × 10^-7^
	-6.218 × 10^-4^	1.98 × 10^-7^
	-6.162 × 10^-4^	2.47 × 10^-7^
	-6.070 × 10^-4^	2.97 × 10^-7^

Where k` is the pseudo first order rate constant.

Regression of (C) versus K` gave equations:

k`=−4.195+847496 C  (r=0.960) for VPk`=−1.305+828048 C  (r=0.9528) for DLTk`=−1.287+735044 C  (r=0.9949) for NCk`=−6.498+1407295 C  (r=0.9963) for FZ

where C is the molar concentration of the drugs.

### Fixed fluorescence method

Reaction times required to reach specific fluorescence of redox reaction for different concentrations of VP, DLT, NC and FZ in the range of 1.50 × 10^-7^ – 2.04 × 10^-7^ M, 4.43 × 10^-8^ – 1.36 × 10^-7^ M, 8.91 × 10^-8^ – 1.77 × 10^-7^ M and 1.48 × 10^-7^ – 2.97 × 10^-7^ M, respectively were recorded. A preselected values of the fluorescence 301, 155, 140 and 181 for VP, DLT, NC and FZ were fixed, respectively and the time was measured in seconds. The reciprocal of time (1/t) versus the initial concentration of drug was plotted. Table 4 and the following equations of the calibration graphs were obtained:

1/t=−1.553×10−3+15762.88 C r=0.9977 for VP1/t=−4.571×10−4+27605.75 C r=0.9972 for DLT1/t=−7.922×10−4+21257.45 C r=0.9999 for NC1/t=−1.573×10−3+16597.49 C r=0.9984 for FZ

where C is the molar concentration of the drugs and t = time in second.

**Table 4:** Application of the fixed fluorescence method in the quantification of the studied drugs by the proposed method

Compound	Time (sec.)	1/t (sec.^-1^)	[drug]

Verapamil HCl	1200	8.33 × 10^-4^	1.50 × 10^-7^
	900	1.11 × 10^-3^	1.71 × 10^-7^
	600	1.67 × 10^-3^	2.04 × 10^-7^
Diltiazem HCl	1200	8.33 × 10^-4^	4.43 × 10^-8^
	600	1.67 × 10^-3^	8.09 × 10^-8^
	300	3.33 × 10^-3^	1.36 × 10^-7^
Nicardipine HCl	900	1.11 × 10^-4^	8.91 × 10^-8^
	600	3.30 × 10^-3^	1.16 × 10^-7^
	300	6.80 × 10^-3^	1.94 × 10^-7^
Flunarizine	1200	8.33 × 10^-3^	1.48 × 10^-7^
	600	1.67 × 10^-3^	1.90 × 10^-7^
	300	3.33 × 10^-3^	2.97 × 10^-7^

### Fixed time method

At a preselected fixed time, which was accurately determined, the fluorescence was measured. Calibration graphs of fluorescence versus initial concentrations of VP, DLT, NC and FZ at fixed times for VP and FZ at 5, 10, 15, 20, 25 minutes, for NC at 5, 10, 15 minutes and for DLT at 5, 10, 15, 20 were established with the regression equations and correlation coefficients assembled in Table 5.

**Table 5:** Application of the fixed time method in the quantification of the studied drugs by the proposed method

Compound	Time (min.)	Regression Equations	Correlation Coefficient

Verapamil HCl	5	F = 5.667 + 2621.429 C	r=0.9991
	10	F = 3.267 + 3048.571 C	r=0.9991
	15	F = 5.467 + 3574.286 C	r=0.9992
	20	F = 14.200 + 3811.429C	r=0.9993
	25	F = 7.133 + 4260.000 C	r=0.9998
Diltiazem HCl	5	F = -2.814 + 2533.721 C	r=0.9991
	10	F = 12.116 + 3952.326 C	r=0.9995
	15	F = 4.535 + 5290.698 C	r=0.9996
	20	F = 9.512 + 7380.233 C	r=0.9998
Nicardipine HCl	5	F = -2.80 + 1554.286 C	r=0.9997
	10	F = 7.80 + 2188.571 C	r=0.9998
	15	F = 3.67 + 2992.857 C	r=0.9999
Flunarizine	5	F = -1.00 + 1510.00 C	r=0.9993
	10	F = -1.20 + 2395.00 C	r=0.9993
	15	F = -4.40 + 2750.00 C	r=0.9994
	20	F = 6.40 + 2865.00 C	r=0.9996
	25	F = 4.60 + 3340.00 C	r=0.9998

It is clear that the slope increases with the time and the most acceptable values of the correlation coefficient (r) was chosen as the most suitable time interval for measurement. As a conclusion, the fixed time method was chosen for quantification because it gives the best correlation coefficient in a reasonable time.

## ANALYTICAL PERFORMANCE

The fluorescence-concentration plots for the studied drugs were linear over the range cited in Table 1. Linear regression analysis of the data gave the following equations:

F=7.133+4260.00 C r=0.9998 for VPF=9.512+7380.23 C r=0.9998 for DLTF=3.67+2992.86 C r=0.9999 for NCF=4.60+3340.00 C r=0.9998 for F

where F is fluorescence intensity, C is the concentration of the drug (μg mL^-1^) and r is correlation coefficient.

The limit of quantification (LOQ) was determined by establishing the lowest concentration that can be measured according to ICH Q2B recommendations (49), below which the calibration graph is non linear and was found to be 0.02, 0.01, 9.76 × 10^-3^, 0.04 μg mL^-1^ for VP, DLT, NC and FZ, respectively. The limit of detection (LOD) was determined by evaluating the lowest concentration of the analyte that can be readily detected and was found to be 6.33 × 10^-3^, 3.10 × 10^-3^, 2.93 × 10^-3^ and 0.012 μg mL^-1^ for VP, DLT, NC and FZ, respectively. LOQ and LOD were calculated according to the following equations (49):

LOQ=10 ó /SLOD=3.3 ó /S

where ó is the standard deviation of the intercept of regression line and S is the Slope of the calibration curve.

The proposed method was evaluated by studying the accuracy as percent relative error and precision as percent relative standard deviation. The results are abridged in Table 1.

## VALIDATION OF THE METHOD

### Linearity

The proposed method was tested for linearity, specificity, precision, and reproducibility. Linear regression equations were obtained. The regression plots showed a linear dependence of FI value on drug concentrations over the range cited in Table 1. The table also clarifies the lower detection limits as well as the slopes and intercepts. Validation of the method was evaluated by statistical analysis of the regression line regarding standard deviation of the residuals (S_y/x_), the intercept (S_a_), and the slope (S_b_). The small values of the figures point out to the low scattering of the points around the calibration curve.

Statistical analysis (50) of the results, obtained by the proposed and the comparison methods (15, 35, 51) using Student’s t-test and variance ratio F-test, shows no significant difference between the performance of the two methods regarding the accuracy and precision, respectively.

### Accuracy and precision

The intra-day precision was evaluated through replicate analysis of pure samples of 0.04, 0.06 and 0.08 μg mL^-1^ VP as a model example. Each concentration was analyzed three times. The mean percentage recoveries are shown in Table 6. The repeatability and reproducibility of the proposed method are fairly good as indicated by small values of standard deviation (SD).

The inter-day precision was evaluated through replicate analysis of pure 0.08 μg mL^-1^ VP on three successive days. The percentage recoveries based on the average of three separate determinations are abridged in Table 6.

**Table 6:** Precision and accuracy of the proposed method for spectrofluorometric determination of pure VP

Regimen	Parameters	Concentration Added (μg/mL)	Concentration Found (μg/mL)	Recovery %

Intra-day		0.040	0.0392	98.00
			0.0395	98.75
			0.0398	99.50
	(x)			98.75
	± S.D.			0.75
		0.060	0.0598	99.67
			0.0603	100.50
			0.0607	101.17
	(x)			100.45
	± S.D.			0.75
		0.080	0.0799	99.88
			0.0789	98.63
			0.0805	100.63
	(x)			99.71
	± S.D.			1.01
Inter-day	1^st^ day	0.080	0.0788	98.50
	2^nd^ day		0.0815	101.88
	3^rd^ day		0.0808	101.00
	(x)			100.46
	± S.D.			1.75

Each result is the average of three separate determinations.

The accuracy of the proposed method was evaluated by analyzing standard solutions of the studied drugs. The results obtained by the proposed method were compared with those given by the comparison methods (15, 16, 35, 51).

### Robustness of the method

The robustness of the method adopted in the proposed method is demonstrated by the constancy of the fluorescence intensity with the minor changes in the experimental parameters such as 5 × 10^-4^ M Ce (IV) volume, 0.5 ± 0.1 mL, 1 ± 0.2 mL, 1.5 ± 0.25 mL and 0.8 ± 0.2 mL for VP, DLT, NC and FZ, respectively and change in the concentration of sulphuric acid, 1.25 ± 0.25 M, 1 ± 0.1 M, 1.5 ± 0.2 M and 1.5 ± 0.25 M for VP, DLT, NC and FZ, respectively. These minor changes that may take place during the experimental operation didn’t affect the fluorescence intensity.

## PHARMACEUTICAL APPLICATIONS

The proposed method was applied to the determination of the studied drugs in their dosage forms. The specificity of the method was investigated by observing any interference encountered from the common excipients, such as talc (20 mg), lactose (15 mg), starch (15 mg), avisil (15 mg), gelatine (0.7 mg) and magnesium stearate (10 mg). These excipients did not interfere with the proposed method (Table 7). The results of the proposed method were compared with those obtained using the comparison methods (15, 16, 35, 51).

Statistical analysis (50) of the results obtained using Student’s *t*-test and variance ratio F-test revealed no significant difference between the performance of the two methods regarding the accuracy and precision, respectively (Table 7).

**Table 7:** Application of the proposed method to the determination of the studied drugs in dosage forms

Compound	Proposed method			Comparison methods (15, 16, 35, 51)
	Concentration taken (μg mL^-1^)	Concentration found (μg mL^-1^)	Recovery %	Recovery %

Isoptin 80 mg tablets^a^	0.06	0.0606	101.00	98.58
(VP HCl 80 mg/tablet)	0.08	0.0809	101.125	99.29
	0.10	0.0996	99.60	99.82
Mean ± S.D.			100.58 ± 0.85	99.23 ± 0.62
Student’s *t-*test			1.734	(2.776)
F-test			1.90	(19.00)
Isoptin retard 240 mg tablets^a^	0.06	0.0604	100.67	99.29
(VP HCl 240 mg/tablet)	0.08	0.0798	99.75	98.23
	0.10	0.0992	99.23	98.19
Mean ± S.D.			99.88 ± 0.73	98.57 ± 0.63
Student’s *t-*test			2.30	(2.776)
F-test			1.38	(19.00)
Verapamil 40 mg tablets^b^	0.06	0.0593	98.83	99.29
(VP HCl 40 mg/tablet)	0.08	0.0795	99.38	98.94
	0.10	0.1007	100.70	98.55
Mean ± S.D.			99.64 ± 0.96	98.93 ± 0.37
Student’s *t-*test			1.20	(2.776)
F-test			6.43	(19.00)
Altiazem Retard 60 mg tablets^c^	0.02	0.0199	99.50	98.72
(DLT HCl 60 mg/tablet)	0.04	0.0406	101.50	100.25
	0.05	0.0507	101.40	100.62
Mean ± S.D.			100.80 ± 1.13	99.86 ± 1.01
Student’s *t*-test			1.070	(2.776)
F-test			1.25	(19.00)
Delay-tiazem SR 90 mg capsules^d^	0.02	0.0197	98.50	97.72
(DLT HCl 90 mg/capsule)	0.04	0.0403	100.75	101.15
	0.05	0.0508	101.60	98.48
Mean ± S.D.			100.28 ± 1.60	99.12 ± 1.80
Student’s *t*-test			0.832	(2.776)
F-test			1.27	(19.00)
Delay-tiazem SR 120 mg capsules^d^	0.02	0.0201	100.50	99.74
(DLT HCl 120 mg/capsule)****	0.04	0.0405	101.25	100.90
	0.05	0.0504	100.80	98.90
Mean ± S.D.			100.85 ± 0.38	99.85 ± 1.00
Student’s *t-*test			1.614	(2.776)
F-test			6.94	(19.00)
Delay-tiazem SR 180 mg capsules^d^	0.02	0.0204	102.00	100.94
(DLT HCl 180 mg/capsule)	0.04	0.0404	101.00	100.00
	0.05	0.0505	101.00	101.13
Mean ± S.D.			101.33 ± 0.58	100.69 ± 0.61
Student’s *t-*test			1.312	(2.776)
F-test			1.11	(19.00)
Pelcard 50 capsules^e^	0.06	0.0603	100.50	100.00
(NC HCl 50 mg/capsule)	0.08	0.0810	101.25	101.39
	0.10	0.1013	101.30	101.45
Mean ± S.D.			101.02 ± 0.45	100.95 ± 0.82
Student’s *t-*test			0.130	(2.776)
F-test			3.32	(19.00)
Sibelium capsules^f^	0.06	0.0594	99.00	100.82
(FZ HCl 5 mg/capsule)	0.08	0.0806	100.75	101.20
	0.10	0.1012	101.20	98.77
Mean ± S.D.			100.32 ± 1.16	100.26 ± 1.31
Student’s *t-*test			0.059	(2.776)
F-test			1.27	(19.00)

Each result is the average of three separate determinations. Figures between parenthesis are the tabulated t and F values, respectively at *p*=0.05 (50).

a Products of the Arab Drug Company, Cairo, Egypt);

b Product of El-Nasr Pharmaceutical Chemical Company (Cairo, Egypt);

c Product of Lusofarmaco, Cairo, Egypt);

d Products of GlaxoWellcome Company, El-Salam City, Cairo, Egypt;

e Product of Global Napi & Makram Mehany, Cairo, Egypt);

f Product of Janssen Cilag Company, Cairo, Egypt, Under License of Janssen Pharmaceutica-Belgium).

## MECHANISM OF THE REACTION

**Figure 7:**
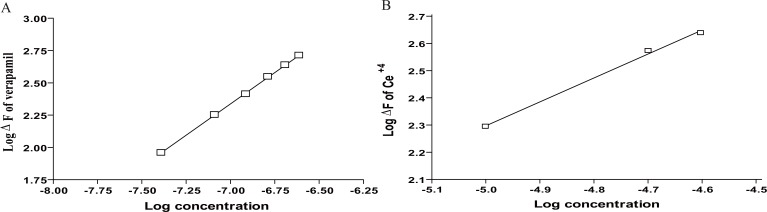
Stoichiometry of the reaction between VP and Ce (IV) adopting limiting logarithmic method. A, Log (VP] vs log ΔF; B, Log (Ce (IV)] vs log ΔF.

The Stoichiometry of the reaction between the studied drugs and cerium (IV) was studied adopting the limiting logarithmic method (53) (Fig. 7). The fluorescence intensities of the reaction product were alternatively measured in the presence of excess Ce (IV) and the studied drugs. Plots of log [drugs] versus log ΔF and log [Ce (IV)] versus log ΔF gave straight lines, the values of the slopes were 0.97: 0.1.60 for VP, 0.99: 1.52 for DLT, 0.97: 1.54 for NC and 0.982: 1.51 for FZ (drug: Ce (IV)). Hence, it is concluded that, the molar reactivity of the reaction is 1: 2 i.e. the reaction proceeds in ratio of 1: 2.

**Figure 8:**
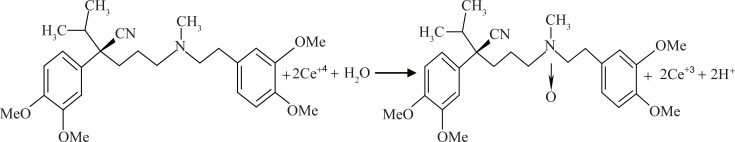
Proposal for the reaction between VP and Ce (IV).

**Figure 9:**
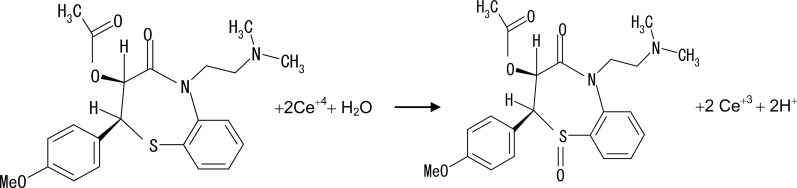
Proposal for the reaction between DLT and Ce (IV).

**Figure 10:**
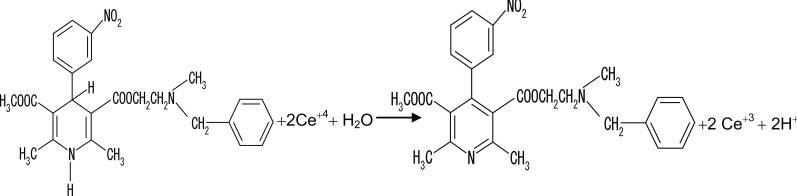
Proposal for the reaction between NC and Ce (IV).

**Figure 11:**
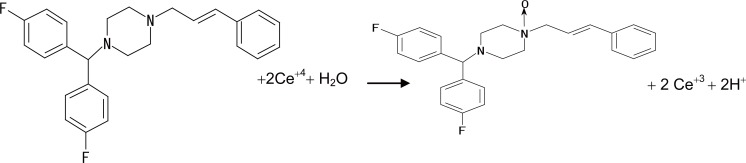
Proposal for the reaction between FZ and Ce (IV).

Based on the above fact and by analogy to previous reports (41), proposals for the reactions between the studied drugs and Ce (IV) shown in the following figures (Figs. 8, 9, 10, 11).

## CONCLUSION

The present work describes a validated spectrofluorometric method for the determination of the studied drugs without interference from common excipients. Hence, it could be applied for the routine quality control of the studied drugs either in bulk or in their corresponding dosage forms. The methodology appears to be straightforward and results are relevant. Another advantage is that, comparing to the existing spectrofluorometric methods, the proposed method is several times more sensitive. From economic point of view, the proposed method is simple, rapid and inexpensive besides the use of water as diluting solvent. So, it is a good alternative to the reported methods and to high cost HPLC methods.
